# Mechanisms of Lymphatic Regeneration after Tissue Transfer

**DOI:** 10.1371/journal.pone.0017201

**Published:** 2011-02-17

**Authors:** Alan Yan, Tomer Avraham, Jamie C. Zampell, Seth Z. Aschen, Babak J. Mehrara

**Affiliations:** The Division of Plastic and Reconstructive Surgery, Department of Surgery, Memorial Sloan-Kettering Cancer Center, New York, New York, United States of America; University of Frankfurt - University Hospital Frankfurt, Germany

## Abstract

**Introduction:**

Lymphedema is the chronic swelling of an extremity that occurs commonly after lymph node resection for cancer treatment. Recent studies have demonstrated that transfer of healthy tissues can be used as a means of bypassing damaged lymphatics and ameliorating lymphedema. The purpose of these studies was to investigate the mechanisms that regulate lymphatic regeneration after tissue transfer.

**Methods:**

Nude mice (recipients) underwent 2-mm tail skin excisions that were either left open or repaired with full-thickness skin grafts harvested from donor transgenic mice that expressed green fluorescent protein in all tissues or from LYVE-1 knockout mice. Lymphatic regeneration, expression of VEGF-C, macrophage infiltration, and potential for skin grafting to bypass damaged lymphatics were assessed.

**Results:**

Skin grafts healed rapidly and restored lymphatic flow. Lymphatic regeneration occurred beginning at the peripheral edges of the graft, primarily from ingrowth of new lymphatic vessels originating from the recipient mouse. In addition, donor lymphatic vessels appeared to spontaneously re-anastomose with recipient vessels. Patterns of VEGF-C expression and macrophage infiltration were temporally and spatially associated with lymphatic regeneration. When compared to mice treated with excision only, there was a 4-fold decrease in tail volumes, 2.5-fold increase in lymphatic transport by lymphoscintigraphy, 40% decrease in dermal thickness, and 54% decrease in scar index in skin-grafted animals, indicating that tissue transfer could bypass damaged lymphatics and promote rapid lymphatic regeneration.

**Conclusions:**

Our studies suggest that lymphatic regeneration after tissue transfer occurs by ingrowth of lymphatic vessels and spontaneous re-connection of existing lymphatics. This process is temporally and spatially associated with VEGF-C expression and macrophage infiltration. Finally, tissue transfer can be used to bypass damaged lymphatics and promote rapid lymphatic regeneration.

## Introduction

Lymphedema is the chronic swelling of an extremity that occurs when the capacity of the lymphatic system to drain protein-rich interstitial fluid is exceeded.[1] In the United States and Western countries this condition occurs most commonly after lymph node resection for the treatment of breast and gynecological cancers.[2], [3], [4] It is estimated that as many as 50% of patients treated with lymph node dissection go on to develop lymphedema, even after more limited surgeries such as sentinel lymph node biopsy.[5], [6], [7] Patients with this complication have a significantly decreased quality of life with frequent infections, decreased range of motion, and a cosmetic deformity that is difficult to conceal.[8] Furthermore, lymphedema results in a nearly $10,000 increase in the two-year treatment cost of breast cancer patients.[9] With over 200,000 new breast cancers diagnosed annually, it is clear that lymphedema is a significant biomedical burden.

Treatment for lymphedema has largely been symptomatic in nature and designed to prevent progression of swelling. Patients are treated with compressive stockings and manual massage to decrease fluid accumulation and encourage drainage of interstitial fluid.[10] These treatments can decrease swelling and discomfort but are time consuming and do not reverse the basic pathology of lymphedema. More importantly, once compression and manual drainage are stopped, lymphedema recurs and in most cases worsens over time.

Some surgeons have attempted to bypass damaged lymphatic channels by interposition of healthy tissues. In fact, a few clinical case studies have shown remarkable improvements in limb edema after tissue transfer with evidence of lymphatic re-routing.[11], [12] Clinical evidence of spontaneous lymphatic regeneration is also noted after microsurgical tissue transfer.[13] In these procedures, composite tissues containing skin and fat are transferred from areas with excess tissues (e.g. abdomen) to replace damaged or surgically resected tissues by reconnecting the arterial and venous vessels using microsurgery. Although the lymphatic vessels are not re-anastomosed, tissue edema spontaneously resolves over a period of 6–8 weeks implying that lymphatic regeneration has occurred. More recently, Tammela *et al.* demonstrated that lymph node transfer combined with VEGF-C administration after lymphadenectomy in mice can promote reconstitution of the deep lymphatic system.[14] Similarly, Kerjaschki *et al.* have demonstrated that the majority of lymphatics (>95%) present in chronically rejected kidney transplants originated from the donor.[15] Thus, transfer of healthy tissues may be a means of replacing or re-routing damaged lymphatic vessels to treat or prevent lymphedema. The purpose of these experiments was to investigate the mechanisms that regulate lymphatic regeneration after tissue transfer.

## Methods

### Mouse tail excision model, surgical preparation, and full-thickness skin grafting

In order to investigate the mechanisms that regulate lymphatic regeneration after tissue transfer, we excised a 2-mm wide, full-thickness area of skin in 10–12 week old female athymic nude mice (nu/nu; NCI-Frederick, Frederick, ME). In addition, without injuring the tail blood supply, the deep lymphatic system was visualized and ligated using a surgical microscope (Leica, StereoZoom SZ-4, Wetzlar, Germany). The defects were then either left open or immediately repaired with full-thickness skin grafts harvested from 10–12 week-old female transgenic mice that express green fluorescent protein (GFP, C57BL/6-Tg(CAG-EGFP)1Osb/J; Jackson Labs, Bar Harbor, ME) or LYVE-1 knockout (B6.129S1-*Lyve^1tm1Lhua^*/J; Jackson Labs, Bar Harbor, ME) mice. The skin graft was secured with 9-0 nylon simple interrupted sutures, and the surgical wound was dressed with Tegaderm (3M, St. Paul, MN) adhered proximally and distally to minimize manipulation of the site by the animal post-operatively. Six to eight animals were used per experimental group. All animal procedures were approved by the Resource Animal Research Center Institutional Animal Care and Use Committee (IACUC; protocol #06-08-018) at Memorial Sloan-Kettering Cancer Center, New York, NY.

### Specimen preparation and histology

Ten-millimeter tail sections centered on the repair site were harvested 2 or 6 weeks after surgery and fixed in 4% paraformaldehyde overnight at 4°C. Specimens were then decalcified in Immunocal (Decal Chemical Corp., Tallman, N.Y.), embedded in paraffin, and sectioned longitudinally (5 µm). Immunohistochemical and immunofluorescent staining were performed as previously described.[16] Lymphatic vessels were identified using antibodies against podoplanin (Abcam, Cambridge, MA) and LYVE-1 (R&D Systems, Minneapolis, MN). Macrophages were identified using antibody against F4/80 (Abcam, Cambridge, MA), and VEGF-C expression was identified using antibody against VEGF-C (Abcam, Cambridge, MA). Immunofluorescent secondary antibodies used were Alexa Fluor 488, 594, and 647 (Invitrogen Molecular Probes, Carlsbad, CA). Three-dimensional reconstructions of 35–45 confocal z-stack tissue sections (40x) were created using the Imaris Software (Bitplane Corp, Zurich, Switzerland). For immunohistochemical staining, secondary antibody from the Vectastain ABC Kit (Vector Laboratories, Burlingame, CA) was used and developed using diaminobenzidine. Negative control sections were incubated with secondary antibody only. Images were obtained using bright-field microscopy (Leica TCS) for immunohistochemistry and confocal microscopy (Leica) for immunofluorescence. Cell and vessel counts were performed in 3-5 high power fields per section (n = 6–8/time point) by two blinded reviewers.

### Tail volume calculation

To evaluate the degree of postoperative acute lymphedema with and without skin graft, tail volumes were measured 1, 2, 4, and 6 weeks after surgery in both groups (n = 6–8 animals/group/time point) by blinded reviewers. Tail circumference was measured at 10-mm intervals starting at the distal margin of the tail excision using a digital caliper, and tail volumes were then calculated using the truncated cone formula: V =  ¼π (C_1_C_2_ + C_2_C_3_ + … +C_7_C_8_) from our previous methods.[16]

### Microlymphangiography

Microlymphangiography was performed prior to sacrifice to evaluate the gross structure of the capillary lymphatics as previously described.[17] Briefly, 15 µL of 2000-kDa dextran solution (10 mg/mL) conjugated to fluorescein isothiocyanate (FITC) was injected approximately 10 mm proximal to the tip of the mouse tail under constant pressure. This molecule is too large to enter blood vessels, but can enter lymphatics which are designed to transport large molecules in interstitial fluid. Capillary lymphatics were then visualized in the tail using the Leica MZFL3 Stereoscope (Wetzlar, Germany). Fluorescent images were obtained at consistent magnification using Volocity software (PerkinElmer, Waltham, MA).

### Lymphoscintigraphy

In an effort to quantify lymphatic transport after surgery with or without skin graft, technetium-99 m sulfur colloid (100-nm particle size; 400-800 µCi in ∼50 µl) was injected intradermally approximately 20 mm from the tip of the mouse tail as previously described.[16] Dynamic planar gamma camera images were acquired for 2.5 hours after injection using an X-SPECT camera (Gamma Medica, Northridge, CA), and region-of-interest analysis was performed to derive the decay-adjusted activity using ASIPro software (CTI Molecular Imaging, Knoxville, TN).

### Dermal thickness calculation

Dermal thickness calculation was performed as previously described.[18] Briefly, longitudinal tail sections were stained with hematoxylin and eosin and visualized using the Mirax Slide Scanner (Carl Zeiss Microimaging, Munich, Germany). A distance of 4 mm was measured distal to the wound edge in standardized longitudinal histological sections and the dermal thickness was measured from the dermal/epidermal junction to the deep muscles (2–4 measurements/animal/group; n = 6–8 animals/group).

### Scar index calculation

Sirius red (Direct Red 80; Sigma, St. Louis, MO) staining was performed to evaluate the degree of fibrosis in the specimens as previously described.[16], [19] Birefringence pattern and hue were evaluated using polarized light microscopy to determine collagen density, pattern of deposition, and maturity. This is based on the fact that normal tissues display fine collagen bundles with a yellow-green birefringence in a random pattern while soft tissue fibrosis results in formation of thicker, parallel collagen bundles with a orange-red birefringence.[20] Scar index is a quantitative analysis of fibrosis calculated by comparing the ratio of orange-red to yellow-green staining using Metamorph Offline software (Molecular Devices Corporation, Sunnyvale, CA) with a minimum of 4–6 sections per animal (n = 6–8 animals/group).

### Statistical Analysis

Statistical analysis was performed using GraphPad Prism software for Windows (GraphPad Software, Inc., San Diego, CA). Comparative analysis between multiple groups was performed using the Kruskal-Wallace test with post hoc tests to compare different groups. Differences between 2 groups were evaluated using the Student's t-test. Mean values and standard deviations are presented with *p*<0.05 considered significant unless otherwise noted.

## Results

### Autogenous tissue lymphangiogenesis is associated with spontaneous reconnection of local lymphatics and infiltration of new lymphatic capillaries

**Figure 1:**
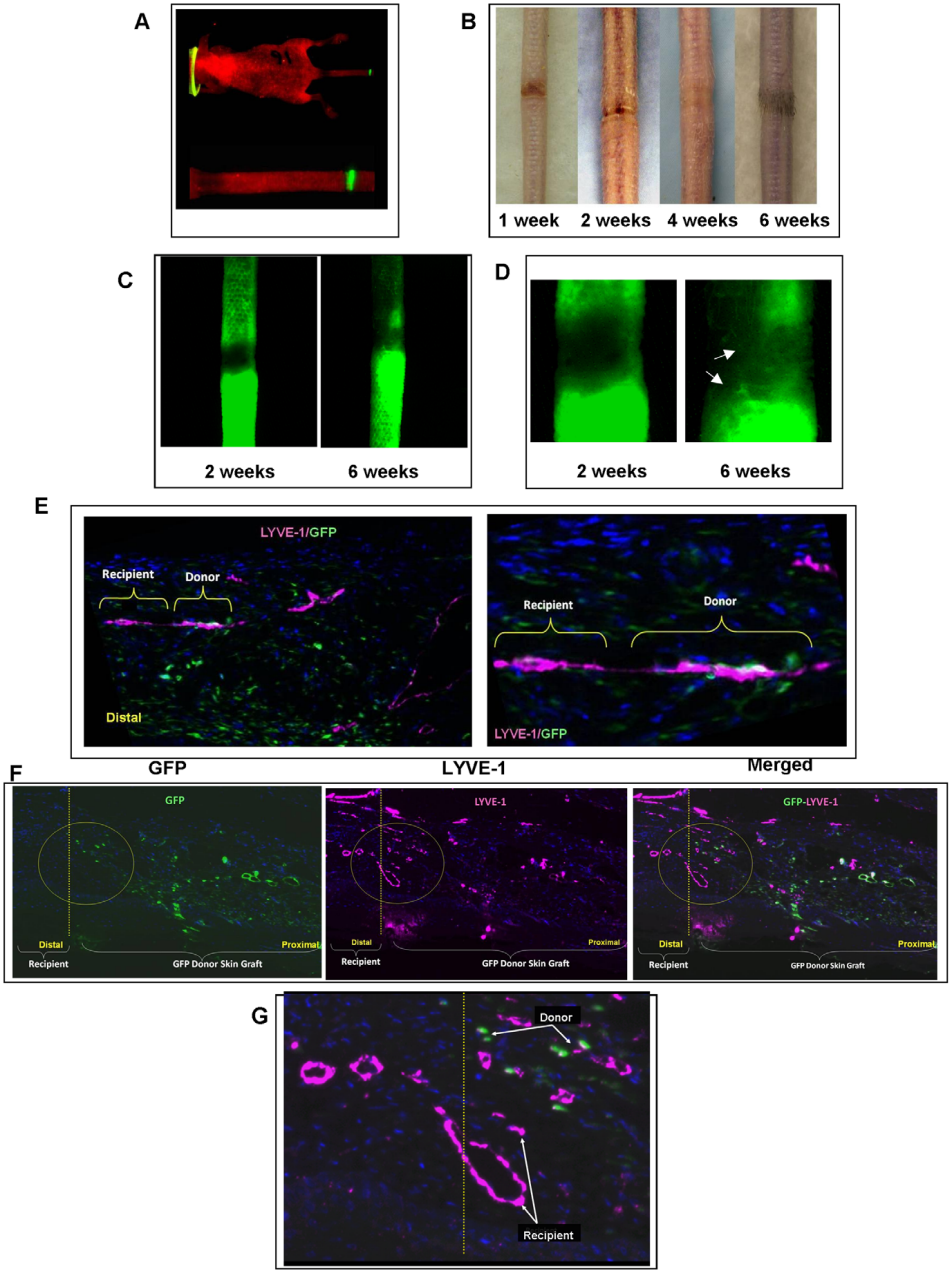
Autogenous tissue lymphangiogenesis is associated with spontaneous reconnection of local lymphatics and infiltration of new lymphatic capillaries. **A.** Skin grafts harvested from GFP transgenic mice express GFP at high levels and for a long period of time. A representative fluorescent picture of a mouse (top) with higher magnification of the tail (bottom) is shown 6 weeks after surgery. The skin-grafted GFP portion of the tail can easily be seen. **B.** Gross photographs of mouse tails treated with skin grafts harvested from GFP transgenic mice. Note the rapid incorporation and ingrowth of hair follicles at the 6-week time point. **C.** Microlymphangiography performed 2 (left) or 6 (right) weeks following skin graft. Distal portion of the tail is shown at the bottom. Note the flow of fluorescent dye by interstitial flow after 2 weeks. In contrast, lymphatic flow can be seen in the skin-grafted area at the 6-week time point. Representative figures of triplicate experiments are shown. **D.** Higher power photograph of microlymphangiography 2 and 6 weeks after surgery demonstrating a few honeycomb-like dermal lymphatics (white arrows) in the skin graft at the 6-week time point. **E.** Representative LYVE-1 (pink) and GFP (green) co-localization in skin-grafted mouse tails 6 weeks after surgery. Low power (5x; left) and high power (20x; right) views are shown. Note connection between GFP^-^ (recipient) and GFP^+^ (donor) lymphatic vessel. **F.** Representative photomicrograph (2x) demonstrating GFP (left), LYVE-1 (middle), and co-localization (right) of skin-grafted mouse tails 6 weeks after surgery. Note ingrowth of GFP^-^/LYVE-1^+^ vessels (yellow circle) from the distal (yellow dotted line) portion of the wound. **G.** High power (20x) view of section shown in **F**. Note the presence of both recipient (GFP^-^/LYVE-1^+^) and donor (GFP^+^/LYVE-1^+^) lymphatics at the distal margin of the wound.

In order to investigate the involvement of recipient and donor cells in spontaneous lymphatic regeneration, the tails of athymic nude mice that underwent full-thickness skin excision were repaired with skin grafts harvested from GFP mice. This allowed us to follow the origin of cells by co-localization of GFP and lymphatic markers. Using this technique, the recipient nude mice lymphatics stained positive for lymphatic markers but negative for GFP. The transplanted tissue lymphatics, in contrast, stained positive for both GFP and lymphatic markers (**Figure 1**). Using fluorescent imaging, we found that high-level GFP expression was maintained in the transplanted skin even 6 weeks after tissue transfer (**Figure 1A**). The transplanted skin grafts healed quickly and with regrowth of hair and skin appendages by 6 weeks indicating that the grafts had become fully incorporated (**Figure 1B**). In addition, return of lymphatic function with transport of fluorescence-labeled colloid could be grossly visualized by microlymphangiography as early as 2 weeks after surgery (**Figure 1C**). This transport was initially driven by interstitial flow (i.e. no discrete lymphatic vessels could be visualized at 2 weeks); however, by 6 weeks honeycomb-like dermal lymphatics were noted in the transferred grafts (primarily at the distal margin) suggesting that the reformed lymphatic vessels are functional (**Figure 1D**). The lack of complete restoration of the honeycomb-like dermal lymphatic architecture in the mid portion of the graft at this time may reflect a delay in dermal lymphatic regeneration. This concept is supported by previous studies demonstrating that honeycomb-like lymphatic vessels that regenerate in the mouse tail after coverage of the wound with collagen gel are not ordinarily visible until day 60 after surgery and even at this time are only seen at the distal margin of the wound.[21], [22]

Confocal co-localization of GFP and LYVE-1 in sections obtained 6 weeks after surgery demonstrated the presence of numerous lymphatic vessels that were comprised of a portion of both recipient (GFP^-^/LYVE-1^+^) and donor (GFP^+^/LYVE-1^+^) lymphatic endothelial cells (**Figure 1E**)**.** We also visualized thin lymphatic bridges connecting donor and recipient lymphatics suggesting that these vessels were spontaneously re-connecting at the edge of the wound. These connections were not frequently noted, however, due to the fact that the sections were performed in the longitudinal plane (i.e. necessitating sectioning at an extremely small/precise plane). In addition to these lymphatics, numerous recipient lymphatic vessels infiltrating the skin graft were noted primarily at the distal edge of the wound, suggesting that lymphatic regeneration of transplanted skin occurs via both spontaneous reconnection and ingrowth of new capillary lymphatic channels (**Figures 1F–G**)**.** Interestingly, at the early time point, qualitatively, the majority of vessels present were GFP^+^/LYVE-1^+^; however, by 6 weeks these vessels represented a relatively small percentage of the total number of lymphatic vessels (**Figure 1F–G**). Precise quantification of donor and recipient vessels was difficult to perform with GFP skin grafts, however, due to the fact that GFP is expressed in all cells and not limited solely to the lymphatics. Due to this limitation, we performed identical operations using skin grafts obtained from LYVE-1 knockout mice.

### Lymphatic regeneration after tissue transfer is associated with infiltration of LYVE-1 positive cells

To further investigate the role of donor and recipient cells in lymphatic regeneration, athymic nude mice underwent full-thickness skin grafting with tissues harvested from LYVE-1 knockout mice. This approach again enabled us to trace the origin of the lymphatic vessels and differentiate between donor and recipient vessels more easily than GFP skin grafts since donor tissues lacked LYVE-1 expression (a molecule that is relatively specific to lymphatic vessels) but expressed podoplanin (podoplanin^+^/LYVE-1^-^), whereas recipient vessels expressed both molecules (podoplanin^+^/LYVE-1^+^).

**Figure 2:**
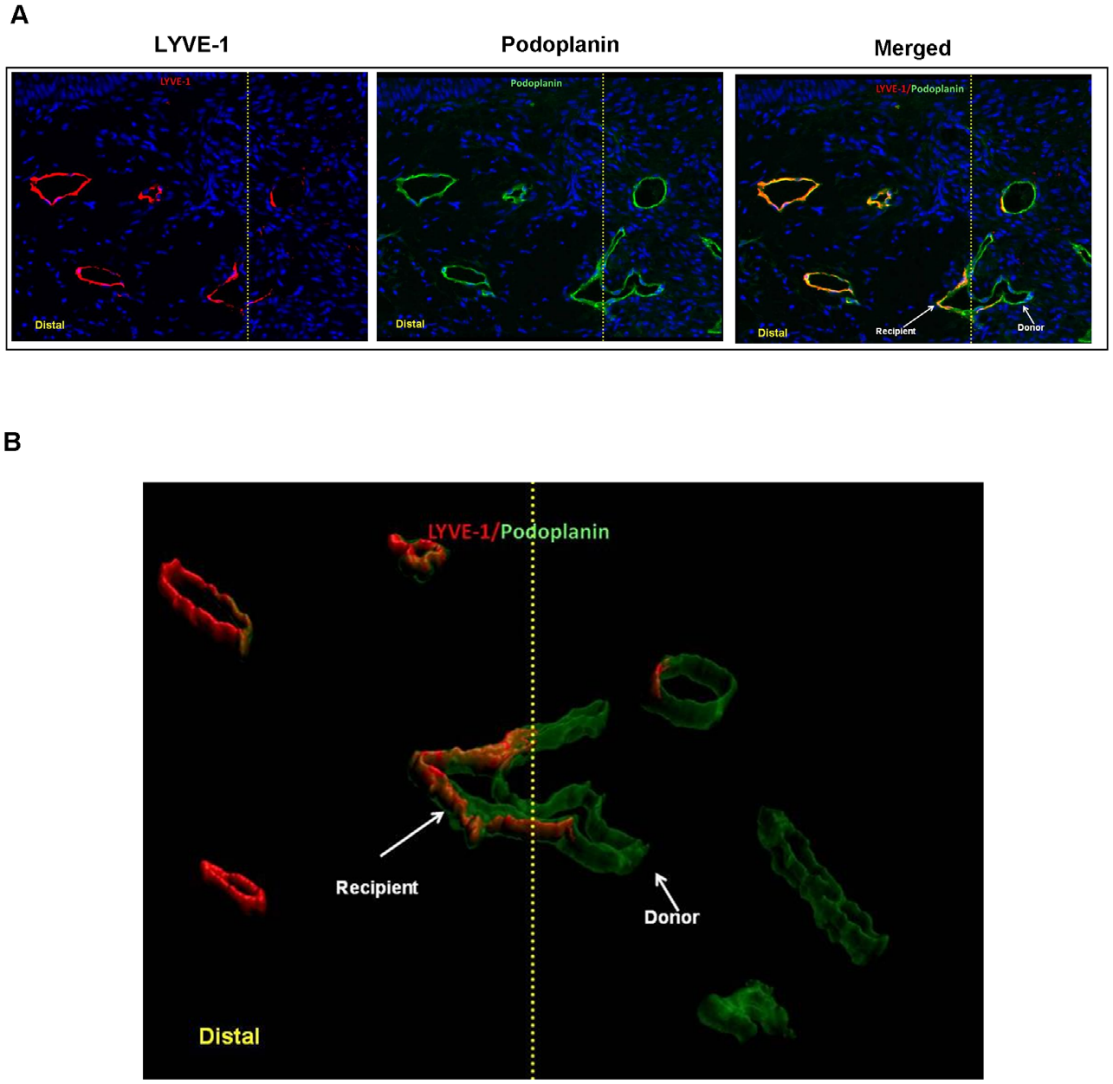
Lymphatic regeneration after tissue transfer occurs in part from spontaneous reconnection of recipient and donor lymphatic vessels. **A.** Co-localization of LYVE-1 (left panel) and podoplanin (middle panel) of mouse tail sections harvested 6 weeks after surgery (40x). Overlay of images demonstrates connection of LYVE-1^+^ (i.e. recipient) and LYVE-1^-^ (i.e. donor) derived podoplanin-stained lymphatics. Yellow line marks the junction of the skin graft and native tail skin. Distal junction of the skin graft and mouse tail is shown to the left. **B.** Three-dimensional rendering of podoplanin/LYVE-1 co-localization at the junction of the skin graft and native tail skin (yellow line). Note connection between LYVE-1^+^ and LYVE-1^-^ lymphatic. Also note presence of both recipient (podoplanin^+/^LYVE-1^+^) and donor (podoplanin^+^/LYVE-1^-^) vessels in the section.

To investigate the relative contribution of donor and recipient lymphatic vessels to lymphatic regeneration in the transplanted skin, immunofluorescence staining with the lymphatic endothelial cell markers LYVE-1 and podoplanin was performed and the number of donor- and recipient-derived lymphatic vessels were evaluated at various regions of the skin graft (distal, middle, and proximal portions) 2 or 6 weeks following surgery (**Figures 2A–B**). This analysis, similar to our GFP/LYVE-1 co-localization, demonstrated connections between LYVE-1^+^ and LYVE-1^-^ lymphatic vessels suggesting that lymphatic vessels from the recipient and donor tissues spontaneously reconnect. This phenomenon is seen in 3-D reconstructions of z-stacked confocal images (**Figure 2B**)**.**

**Figure 3:**
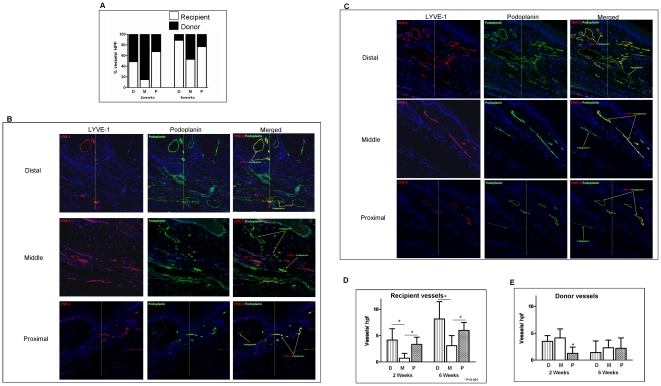
Both reconnection and infiltration of LYVE-1^+^ lymphatic vessels contribute to lymphatic regeneration after tissue transfer. **A.** Bar graph depicting source of lymphatic vessels in various regions of the skin-grafted tissues 2 or 6 weeks after surgery. Origin of lymphatic vessels in various regions of the skin graft 2 or 6 weeks after surgery was determined using podoplanin/LYVE-1 co-localization in skin grafts harvested from LYVE-1 knockout mice and transferred to nude mice. Recipient lymphatic vessels were identified as podoplanin^+^/LYVE-1^+^ (white bars) while donor lymphatics were identified as podoplanin^+^/LYVE-1^-^ (black bars). Recipient-derived lymphatic vessels were noted primarily in the distal and proximal portions of the graft at the 2-week time point and became more uniform in distribution by 6 weeks. Increased number of recipient vessels in the peripheral regions of the graft at the 6-week time point resulted in a relative decrease in the percentage of donor-derived lymphatics at this time. **B, C.** Representative images (10x) of LYVE-1 (red) and podoplanin (green) staining of the distal portion of the graft 2 (**B**) and 6 (**C**) weeks after surgery. Yellow line marks the junction of the skin graft (located to the right) and recipient (located to the left) tissues. **D.** Recipient lymphatic vessels (podoplanin^+^/LYVE-1^+^) in various regions of the skin graft 2 and 6 weeks after skin grafting. Note that the numbers of recipient vessels in the distal (D) and proximal (P) portions of the tail are significantly greater than the number of recipient lymphatics in the middle (M) portion of the graft at both the 2- and 6-week time points indicating ingrowth of vessels from the periphery (*p<0.05). In addition, the number of recipient lymphatics in various regions of the skin graft increased significantly when comparing 2- and 6-week time points indicating ingrowth of recipient vessels. **E.** Donor lymphatic vessels (podoplanin^+^/LYVE-1^-^) in various regions of the skin graft 2 and 6 weeks after skin grafting (*p<0.05). The number of donor vessels remained essentially unchanged at both time points indicating that donor lymphatics persist over time and do not proliferate after tissue transfer.

Quantification of donor and recipient lymphatic vessels also suggested that both reconnection and infiltration of recipient-derived lymphatics contribute to lymphatic regeneration in the transplanted skin. Two weeks following tissue transfer, 52% of the vessels in the distal margin and 37% of those at the proximal margin were donor-derived (i.e. podoplanin^+^/LYVE-1^-^). In contrast, 92% of the lymphatic vessels in the middle portion were donor-derived. By 6 weeks, the vast majority (>90%) of lymphatic vessels present in the distal and proximal margins were of recipient origin. Similarly, the number of recipient vessels in the middle portion also increased such that the distribution of these vessels with donor-derived lymphatics was 1∶1. These differences in the distribution of the lymphatic vessels were primarily due to the ingrowth of new lymphatics since the number of recipient vessels in the later time points increased substantially while the number of donor vessels (similar to our qualitative observations in the GFP experiment) decreased slightly, though not statistically significant (**Figures 3D–E**). There were significantly more recipient-derived vessels at the distal and proximal margins as compared to the central aspect of the graft suggesting ingrowth of vessels from the periphery of the transferred tissues (8.2 and 6.0 vs. 3.1; *p*<0.05). Taken together these findings suggest that donor-derived lymphatic vessels persist over time and that transferred tissues become further infiltrated by lymphatic vessels originating from the recipient beginning at the distal and proximal edges and infiltrating towards the center of the graft.

### Lymphatic regeneration after tissue transfer is associated with expression of VEGF-C and infiltration of macrophages

**Figure 4:**
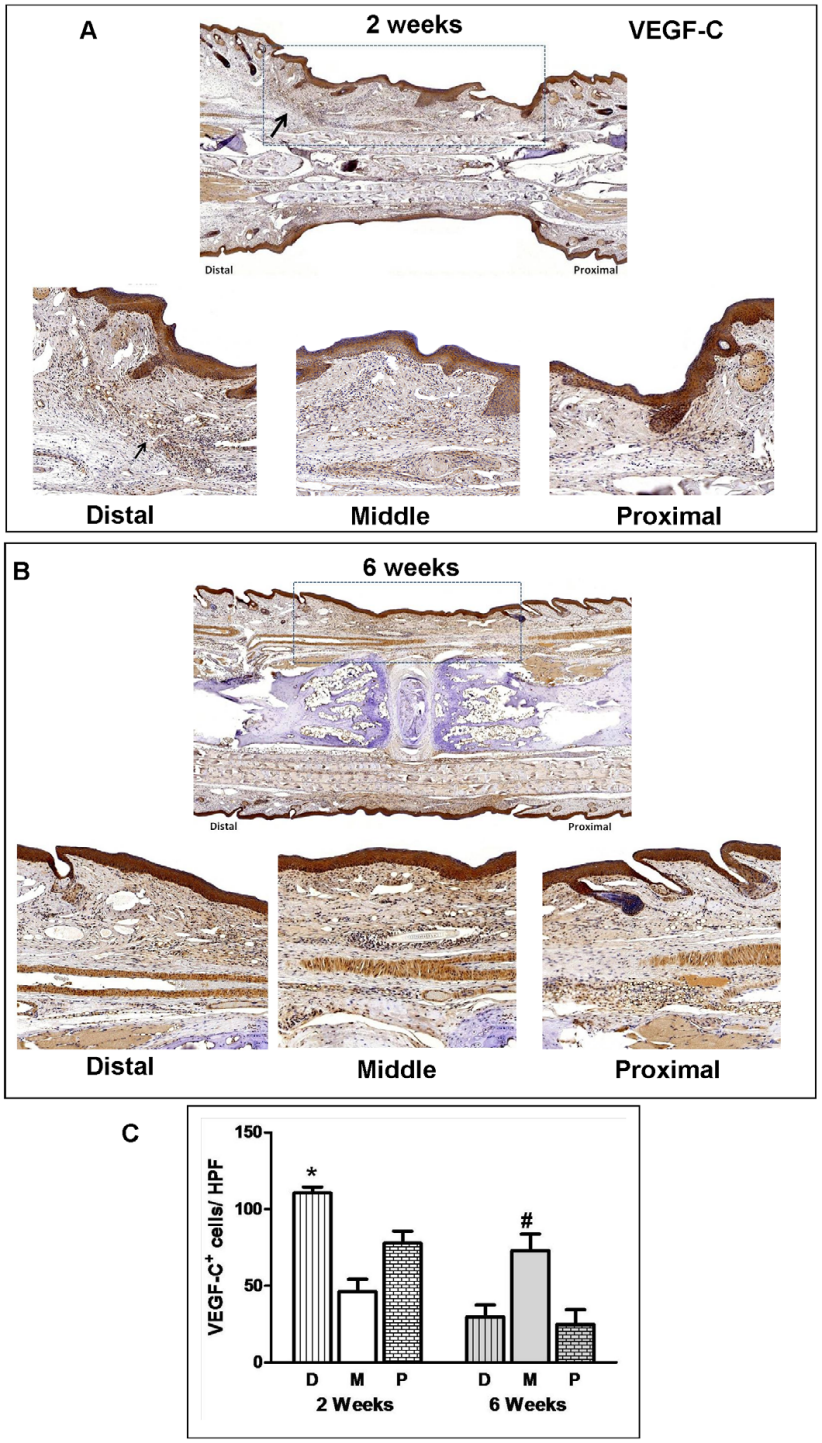
Lymphatic regeneration after tissue transfer is associated with expression of VEGF-C. **A.** VEGF-C expression in skin-grafted tails 2 weeks after surgery. Representative low power (2x; upper panel) photomicrographs encompassing the skin-grafted area and distal/proximal portions of the recipient mouse-tail are shown. High power (20x) views of the distal and proximal junctions between recipient tissues and skin grafts are shown below. Black arrow shows large number of VEGF-C^+^ cells in the distal junction. Dashed box delineates skin-grafted area. **B.** VEGF-C expression in skin-grafted tails 6 weeks after surgery. Representative low power (2x; upper panel) and high power (20x) photomicrographs encompassing the skin-grafted area and distal/middle/proximal portions of the recipient mouse tails are shown. Dashed box delineates skin-grafted area. Note small amount of wound/skin graft contracture after repair. **C.** Cell counts per high power field of VEGF-C^+^ cells in the various tail regions (D = distal, M = middle, P = proximal) 2 and 6 weeks after surgery. Cell counts are means ± SD of at least 4 high power fields/mouse/time point. At least 6 mice were analyzed in each group (*p<0.05; *#*<0.01).

VEGF-C is a critical regulator of lymphatic regeneration during adult lymphangiogenesis.[17] Therefore, we investigated the pattern of VEGF-C expression after tissue transfer using immunohistochemical staining. Two weeks after surgery, VEGF-C expression was highest at the periphery of the wounds with the distal wound margin displaying significantly more staining of VEGF-C^+^ cells/hpf than either the middle or proximal portion (110±4 in distal portion vs. 46±8 in middle portion; *p*<0.05; **Figures 4A, C**). VEGF-C staining at the peripheral margins of the grafts was associated with a modest inflammatory infiltrate into the skin graft. By 6 weeks, VEGF-C staining was primarily localized to the central region of the skin graft (73±11 in the central portion vs. 30±8 or 25±10 in proximal or distal portion; *p*<0.05; **Figures 4B–C**). This expression pattern corresponded to the infiltration of lymphatic vessels into the graft beginning at the peripheral margins and extending into the central portion.

**Figure 5:**
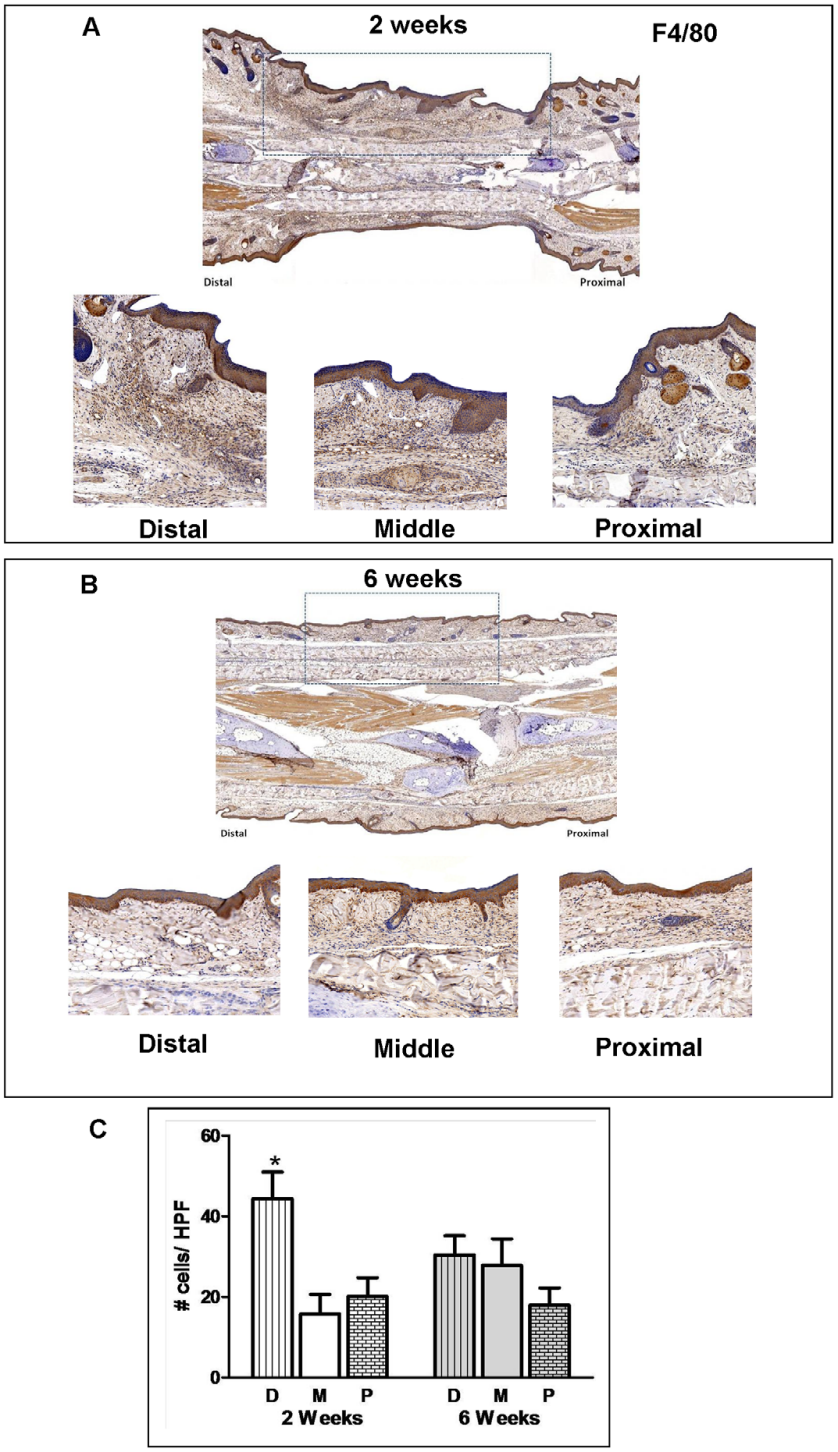
Lymphatic regeneration after tissue transfer is associated with infiltration of macrophages. **A.** F4/80 localization in skin-grafted tails 2 weeks after surgery. Representative low power (2x; upper panel) photomicrographs encompassing the skin-grafted area and distal/proximal portions of the recipient mouse-tail are shown. High power (20x) views of the distal and proximal junctions between recipient tissues and skin grafts are shown below. Dashed box delineates skin-grafted area. **B.** F4/80 localization in skin-grafted tails 6 weeks after surgery. Representative low power (2x; upper panel) and high power (20x) photomicrographs encompassing the skin-grafted area and distal/proximal portions of the recipient mouse tails are shown. Dashed box delineates skin-grafted area. Note small amount of wound/skin graft contracture after repair. **C.** Cell counts per high power field of F4/80^+^ cells in various regions of the tail 2 and 6 weeks after surgery. Cell counts are means ± SD of at least 4 high power fields/mouse/time point. At least 6 mice were analyzed in each group (*p<0.05).

**Figure 6:**
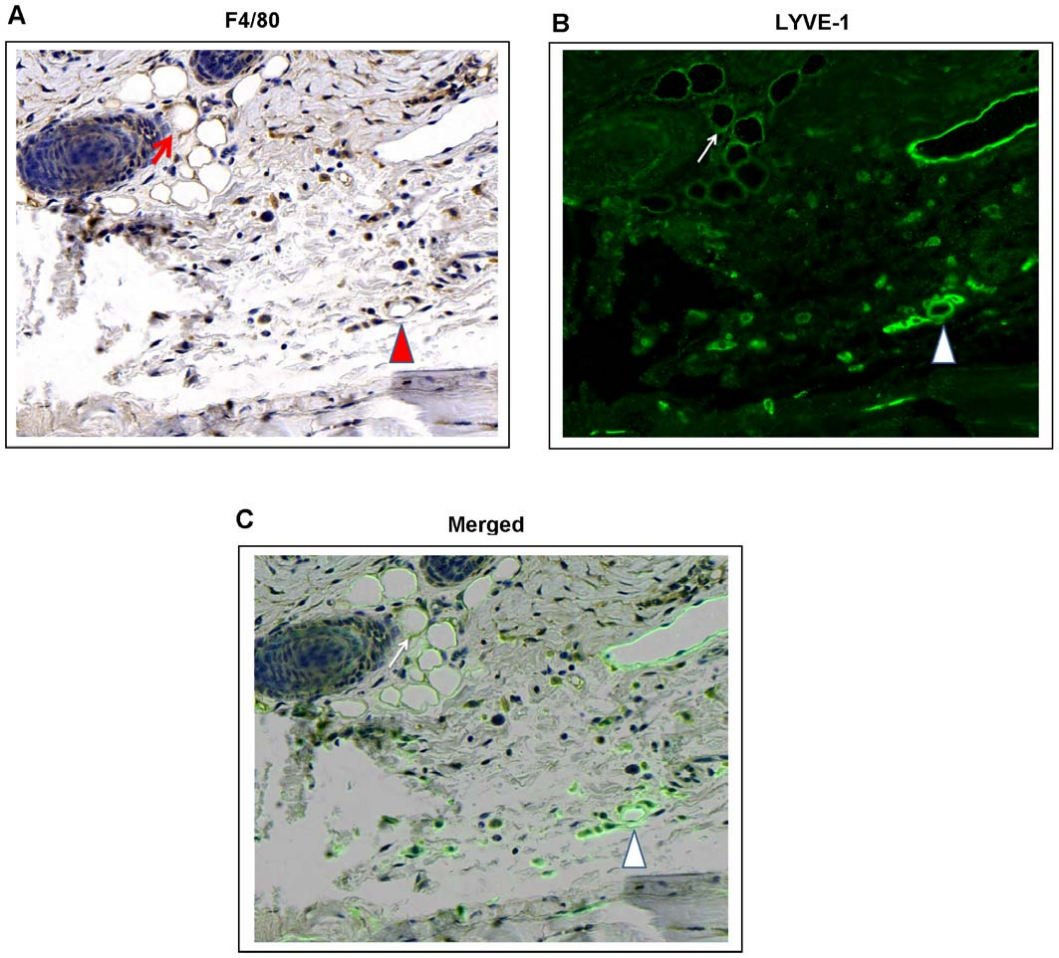
Recipient-derived macrophages express the lymphatic endothelial cell marker LYVE-1 after tissue transfer. **A, B, C.** Representative photomicrograph (40x) demonstrating co-localization of F4/80 (A; brown immunostaining) and LYVE-1 (B; green fluorescent stain) in skin-grafted mouse tail sections 2 weeks after surgery. Arrows show co-localization of F4/80 and LYVE-1 in lymphatic capillaries. Also note scattered F4/80^+^/LYVE-1^+^ cells in the tail section. F4/80^-^/LYVE-1^+^ capillaries can also be appreciated (upper right corner).

Macrophages are critical cells that regulate wound repair and produce VEGF-C during lymphatic regeneration in the mouse tail. In addition, they are thought to directly contribute to lymphangiogenesis by trans-differentiating into lymphatic endothelial cells.[23] Immunohistochemical staining with F4/80 revealed a similar expression pattern as that of VEGF-C with a large number of F4/80^+^ cells localized at the periphery of the graft at the 2 week time point (**Figures 5A, C**)**.** Similar to our findings of VEGF-C expression, the greatest number of macrophages was noted at the distal margin of the wound (44±7 in distal portion vs. 16±5 or 20±5 in middle or proximal areas; *p*<0.05). Co-localization of F4/80 and LYVE-1 suggested that some of the newly formed lymphatic channels in the skin graft may have formed as a result of trans-differentiation of macrophages into lymphatic endothelial cells (**Figure 6**)**.** In addition, scattered, isolated F4/80^+^/LYVE-1^+^ cells that had not formed tubules could be seen within the wound sections. Newly formed lymphatics with trans-differentiating macrophages were recipient-derived since they also expressed LYVE-1. By 6 weeks, the number of macrophages had decreased in the distal margin of the graft and a more uniform distribution was noted throughout the grafted area (**Figures 5B-C**). In addition, we could not find any F4/80^+^/LYVE-1^+^ vessels at this time point indicating loss of F4/80 expression with vessel remodeling (not shown).

### Spontaneous regeneration of lymphatics after tissue transfer can be used to bypass damaged lymphatics

**Figure 7:**
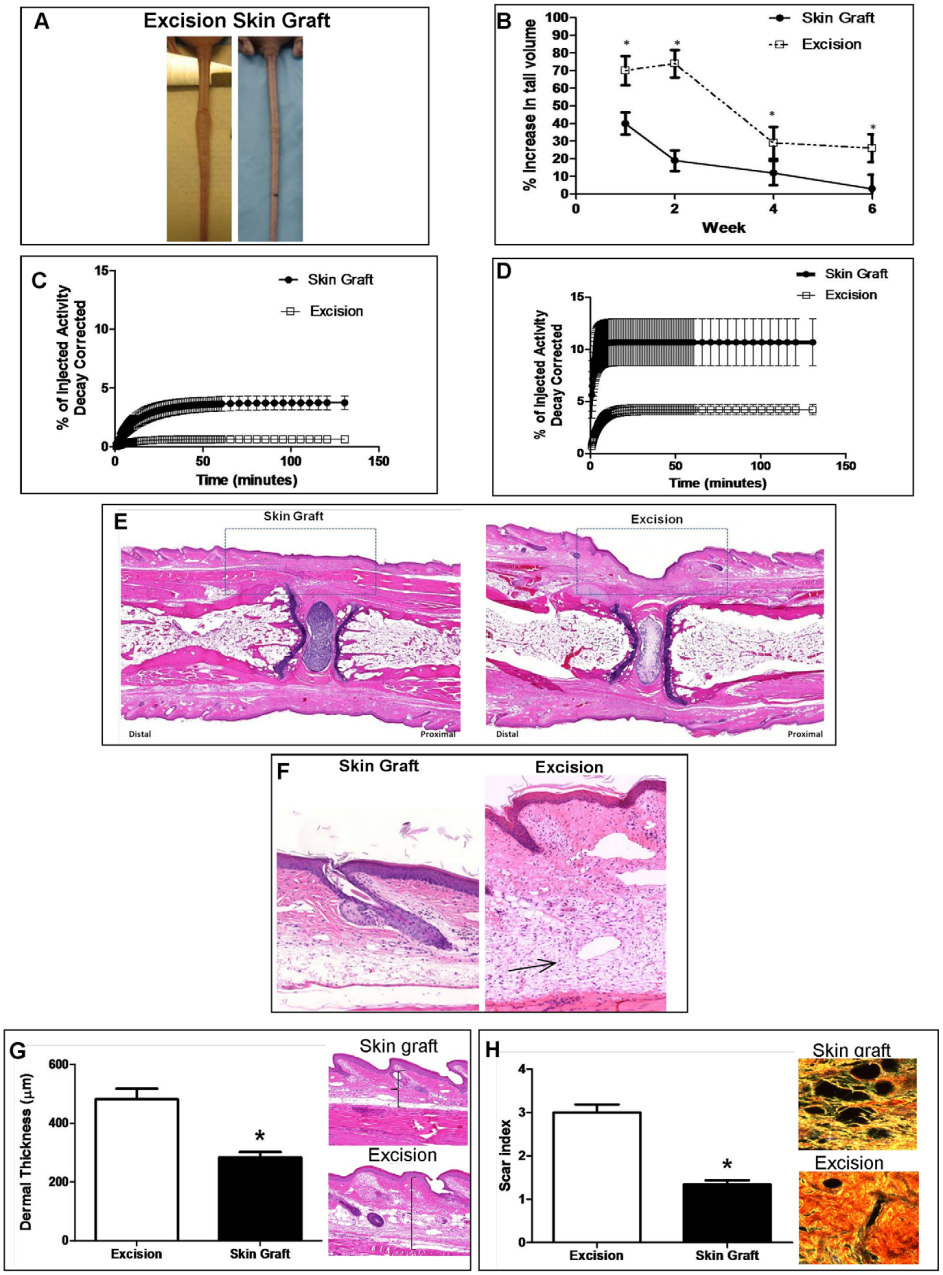
Spontaneous regeneration of lymphatics after tissue transfer can be used to bypass damaged lymphatics. **A.** Gross photographs comparing nude mice that had undergone tail excision with (right) and without (left) skin grafting are shown 6 weeks after surgery. Note obvious difference in tail swelling. **B.** Tail volume measurements in nude mice that had undergone tail excision with or without skin grafting. Data are presented as percent change from baseline (i.e. preoperatively) with mean ± SD (*p<0.05). **C, D.** Representative lymphoscintigraphy of nude mice that had undergone tail excision with or without skin grafting. **E.** Representative photomicrograph (5x) of H&E stained tails sections from nude mice treated with (left) or without (right) 6 weeks after surgery. Dashed box delineates area of skin graft. Note decreased inflammation (cellularity) and dermal thickness in skin-grafted mice distal (to the left) of the wound. **F.** High power (40x) photomicrographs of tail skin harvested 5 mm distal to the excision site. Note decreased cellularity in skin-grafted section (left) as compared with excision section (right; arrow). Also note decreased dermal thickness. **G.** Dermal thickness measurements and representative figures (40x) in nude mice that had undergone tail excision with or without skin grafting 6 weeks following surgery (*p<0.05). **H.** Scar index measurements in tail tissues localized just distal to the site of lymphatic injury 6 weeks after treatment with excision with or without skin grafting. Representative Sirius red birefringence images are shown to the right. Orange-red is indicative of scar; yellow-green is consistent with normal (i.e. non-fibrosed) tissue (*p<0.01).

In order to determine if spontaneous regeneration of lymphatics can be used to bypass surgically damaged lymphatic vessels, we compared lymphatic function in mice that underwent skin excision with animals that had skin excision and repair with full-thickness skin grafting. Evaluation of animals grossly demonstrated marked differences in tail swelling at all time points evaluated (**Figure 7A**). These findings were corroborated by tail volume calculation demonstrating significantly higher tail volumes in excision-only animals at every time point evaluated (**Figure 7B**). Skin-grafted animals had a 4-fold decrease in tail swelling at the 2 week time point (19±5.9% vs. 74±7.8%, *p*<0.05) and their tail volumes returned to baseline 6 weeks after surgery. In contrast, excision-only animals demonstrated a persistent increase even 6 weeks postoperatively (26±8%, *p*<0.01). The differences in tail volumes observed in our study were associated with significantly improved lymphatic function as assessed by lymphoscintigraphy (**Figures 7C–D**). At both the 2 and 6 week time points, the skin-grafted animals demonstrated a significantly increased total uptake of Tc^99^ in the lymph nodes at the base of the tail (3.74% vs. 0.61% at 2 weeks; 10.70% vs. 4.23% at 6 weeks; *p*<0.01 for both time points). In addition, the lymphatic uptake occurred more rapidly as reflected by a more rapid increase in the slope of the asymptotic curve.

Histological analysis of tail sections 6 weeks after surgery demonstrated markedly decreased inflammatory cell infiltrate and dermal thickness in skin-grafted animals as compared to excision-only controls (**Figures 7E–F**). Far fewer inflammatory cells were noted qualitatively in the skin-grafted animals as compared with excision only animals. This was most readily apparent in the portions of the tail distal to the skin excision site (**Figure 7F**). Skin-grafted animals had a 40% decrease in dermal thickness (284±59 µm vs. 482±62 µm, *p*<0.05) and more normal dermal architecture in the regions of the tail localized distal to the site of lymphatic injury (**Figure 7G**). Furthermore, skin-grafted animals demonstrated significantly decreased tissue fibrosis in these regions (**Figure 7H**) with a 54% decrease in the scar index (1.39±0.29 vs. 3.00±0.52, *p*<0.01). Together, these findings support the hypothesis that spontaneous regeneration of lymphatic vessels in autogenous tissues can bypass damaged lymphatic channels resulting in rapid lymphatic repair and markedly improved lymphatic function.

## Discussion

Lymphedema is a debilitating disorder that occurs commonly after lymph node dissection for cancer treatment. Despite its common occurrence and profound impact on quality of life, there is no effective surgical cure. Although clinical reports have shown that autogenous tissue transfer (i.e. transfer of healthy tissues) can bypass damaged lymphatics and improve lymphedema, the precise mechanisms governing lymphatic regeneration in these circumstances remain unknown. The findings of the current study suggest that lymphatic regeneration after tissue transfer occurs as a result of spontaneous reconnection of existing lymphatics as well as ingrowth of new lymphatic vessels from the recipient. In addition, our studies support the hypothesis that these processes occur in the setting of VEGF-C expression and recipient-derived macrophage infiltration beginning at the periphery of the graft. Similar findings have been reported in blood vessel regeneration in skin grafts.[24]

*De novo* lymphangiogenesis has been studied in a variety of human diseases from chronic renal transplant rejection to tumor metastasis. The role of recipient and donor tissues in the regulation of lymphatic regeneration has only been evaluated in a few studies, however. Kerjaschki *et al.* demonstrated that the majority (>95%) of lymphatics in the transplanted kidney are donor-derived while a relatively small number of lymphatic vessels (4.5%) in these tissues were of recipient origin.[15] These results are in contrast to our finding that by 6 weeks following skin grafting, the majority of lymphatic vessels present in the transplanted tissues were of recipient origin (**Figure 3A**)**,** indicating that inosculation with ingrowth of new lymphatic channels is a critical mechanism regulating lymphatic regeneration after skin/subcutaneous tissue transfer but not in solid organ transplantation. Although spontaneous reconnection of existing lymphatics occurs in transplanted skin/subcutaneous tissues, this mechanism is the primary mechanism regulating lymphatic regeneration after solid organ transplantation. It is possible that ingrowth of lymphatic vessels in kidney transplants from surrounding tissues is actively inhibited by the presence of the kidney capsule. Indeed, fibrous capsule and fascial tissues have long been considered to be important barriers to lymphatic tumor metastasis and are used routinely as surgical landmarks during oncological procedures. Alternatively, differences in lymphatic regeneration in solid organs as compared to skin and subcutaneous tissues may reflect volumetric differences in tissues transferred since skin grafts are relatively low volume (and therefore easily infiltrated by surrounding tissues) whereas solid organ transplantation involves a much larger volume of tissues that allows ingrowth of lymphatics only at the periphery of the tissues. Finally, in our model the skin graft was transferred as an avascular tissue with infiltration of local blood vessels and eventual incorporation into the recipient tissues. Kidney transplantation is performed by reconnecting the vascular supply of the tissues thereby minimizing the need for vessel ingrowth.

Previous studies have demonstrated that lymphangiogenesis in the mouse tail model is dependent on gradients of interstitial flow and expression of VEGF-C.[17], [21] These studies have shown that gradients of VEGF-C expression coordinate and regulate lymphatic endothelial cell migration and differentiation resulting in tubule formation and lymphatic regeneration in a distal to proximal direction. In the current study, we also found that 2 weeks following surgery, VEGF-C expression was localized to the peripheral edges of the skin graft especially in the distal margin. These expression patterns correlated with the ingrowth of new lymphatic vessels from the recipient into the donor tissues. Contrary to previous studies with lymphatic regeneration in collagen gel scaffolds, however, we also found lymphatic ingrowth from the proximal aspect of the wound albeit at a slightly slower rate, indicating that lymphatic regeneration across a tissue graft occurs as a result of coordinated lymphatic ingrowth from both margins of the wound. This finding may be due to a higher potential for lymphatic transport via interstitial fluid flow in skin grafts as compared to acellular scaffolds such as type I collagen. Alternatively, it is likely that the skin graft becomes incorporated into recipient tissues more quickly and efficiently than lymphatic/tissue regeneration that occurs in collagen gel scaffolds resulting in improved interstitial flow and expression of VEGF-C even in the proximal portion of the graft.

In the current study we found that by 6 weeks after surgery, VEGF-C expression was highest within the center of the transplanted tissues. This response correlated with the patterns of lymphatic vessel regeneration after tissue transfer, beginning from distal and proximal margins and invading into the center of the donor tissues, implying that lymphatic regeneration occurs as a consequence of VEGF-C expression within the graft. This finding is intriguing and is supported by previous studies demonstrating improved lymphangiogenesis in surgical skin flaps after adenoviral VEGF-C transfer.[25] However, in contrast to these previous studies, we found that endogenous expression of VEGF-C is adequate for promoting rapid and efficient lymphatic regeneration after tissue transfer suggesting that gene therapeutic interventions to augment lymphangiogenic cytokine expression are not necessary for lymphatic regeneration in uncomplicated tissue transfer or surgical interventions.

Macrophages are critical regulators of lymphangiogenesis.[26], [27] Macrophage expression of VEGF-C is a major regulator of lymphangiogenesis in tumors and during inflammation after kidney transplantation or corneal injury.[27], [28] In addition, macrophages are thought to directly contribute to lymphatic vessel formation by trans-differentiating into lymphatic endothelial cells.[29] Our study showed that macrophages served a dual role in lymphatic regeneration after tissue transfer. Immunohistochemical localization of VEGF-C and F4/80 demonstrated that the patterns of VEGF-C expression correlated with accumulation of F4/80^+^ cells (**Figures 3** **,** **4**), supporting the hypothesis that macrophages play a critical role in VEGF-C expression during incorporation and lymphatic regeneration of autogenous tissues. In addition, we found that recipient-derived macrophages may directly contribute to lymphatic formation at the peripheral edges of the graft in the early time period by demonstrating F4/80^+^/LYVE-1^+^ tubular structures at the 2 week time point. Similar to previous studies on inflammatory lymphangiogenesis, these newly formed vessels likely underwent remodeling with loss of F4/80 expression at the later time point since no double positive lymphatic vessels could be identified in sections obtained from animals sacrificed 6 weeks after surgery.[29]

Perhaps the most significant finding of our study was the fact that autogenous tissue transplantation could be used to rapidly bypass damaged lymphatic vessels with resultant restoration of lymphatic flow. Skin-grafted animals had marked reductions in tail swelling and significantly improved lymphatic function, with significant decreases in dermal thickness and tissue fibrosis. The finding that tissue transfer can bypass damaged lymphatic vessels is supported by anecdotal surgical reports demonstrating that tissue transfer can aid in treatment of some patients with lymphedema and provides a mechanism for this observation. Our findings suggest that improvements in extremity swelling occur as a result of both spontaneous lymphatic regeneration and reconnection of local and transferred lymphatic vessels thereby effectively bypassing lymphatic channels damaged during lymphadenectomy. These findings provide a rationale for the development of tissue engineered lymphatic constructs designed specifically for this purpose and may represent a novel means of treating patients with lymphedema.

## References

[1] Rockson SG (2006). Lymphedema.. Curr Treat Options Cardiovasc Med.

[2] Armer J, Fu MR, Wainstock JM, Zagar E, Jacobs LK (2004). Lymphedema following breast cancer treatment, including sentinel lymph node biopsy.. Lymphology.

[3] Beesley V, Janda M, Eakin E, Obermair A, Battistutta D (2007). Lymphedema after gynecological cancer treatment: prevalence, correlates, and supportive care needs.. Cancer.

[4] Hayes SC, Janda M, Cornish B, Battistutta D, Newman B (2008). Lymphedema after breast cancer: incidence, risk factors, and effect on upper body function.. J Clin Oncol.

[5] Petrek JA, Senie RT, Peters M, Rosen PP (2001). Lymphedema in a cohort of breast carcinoma survivors 20 years after diagnosis.. Cancer.

[6] McLaughlin SA, Wright MJ, Morris KT, Giron GL, Sampson MR (2008). Prevalence of lymphedema in women with breast cancer 5 years after sentinel lymph node biopsy or axillary dissection: objective measurements.. J Clin Oncol.

[7] McLaughlin SA, Wright MJ, Morris KT, Sampson MR, Brockway JP (2008). Prevalence of lymphedema in women with breast cancer 5 years after sentinel lymph node biopsy or axillary dissection: patient perceptions and precautionary behaviors.. J Clin Oncol.

[8] Ahmed RL, Prizment A, Lazovich D, Schmitz KH, Folsom AR (2008). Lymphedema and quality of life in breast cancer survivors: the Iowa Women's Health Study.. J Clin Oncol.

[9] Shih YC, Xu Y, Cormier JN, Giordano S, Ridner SH (2009). Incidence, treatment costs, and complications of lymphedema after breast cancer among women of working age: a 2-year follow-up study.. J Clin Oncol.

[10] Hinrichs CS, Gibbs JF, Driscoll D, Kepner JL, Wilkinson NW (2004). The effectiveness of complete decongestive physiotherapy for the treatment of lymphedema following groin dissection for melanoma.. J Surg Oncol.

[11] Slavin SA, Van den Abbeele AD, Losken A, Swartz MA, Jain RK (1999). Return of lymphatic function after flap transfer for acute lymphedema.. Ann Surg.

[12] Classen DA, Irvine L (2005). Free muscle flap transfer as a lymphatic bridge for upper extremity lymphedema.. J Reconstr Microsurg.

[13] Anthony JP, Foster RD, Price DC, Mahdavian M, Inoue Y (1997). Lymphatic regeneration following microvascular limb replantation: a qualitative and quantitative animal study.. J Reconstr Microsurg.

[14] Tammela T, Saaristo A, Holopainen T, Lyytikka J, Kotronen A (2007). Therapeutic differentiation and maturation of lymphatic vessels after lymph node dissection and transplantation.. Nat Med.

[15] Kerjaschki D, Huttary N, Raab I, Regele H, Bojarski-Nagy K (2006). Lymphatic endothelial progenitor cells contribute to de novo lymphangiogenesis in human renal transplants.. Nat Med.

[16] Clavin NW, Avraham T, Fernandez J, Daluvoy SV, Soares MA (2008). TGF-beta1 is a negative regulator of lymphatic regeneration during wound repair.. Am J Physiol Heart Circ Physiol.

[17] Rutkowski JM, Boardman KC, Swartz MA (2006). Characterization of lymphangiogenesis in a model of adult skin regeneration.. Am J Physiol Heart Circ Physiol.

[18] Avraham T, Daluvoy S, Zampell J, Yan A, Haviv YS (2010). Blockade of Transforming Growth Factor-{beta}1 Accelerates Lymphatic Regeneration during Wound Repair.. Am J Pathol.

[19] Pierard GE (1989). Sirius red polarization method is useful to visualize the organization of connective tissues but not the molecular composition of their fibrous polymers.. Matrix.

[20] Ehrlich HP, Desmouliere A, Diegelmann RF, Cohen IK, Compton CC (1994). Morphological and immunochemical differences between keloid and hypertrophic scar.. Am J Pathol.

[21] Boardman KC, Swartz MA (2003). Interstitial flow as a guide for lymphangiogenesis.. Circ Res.

[22] Goldman J, Conley KA, Raehl A, Bondy DM, Pytowski B (2007). Regulation of lymphatic capillary regeneration by interstitial flow in skin.. Am J Physiol Heart Circ Physiol.

[23] Maruyama K, Ii M, Cursiefen C, Jackson D, Keino H (2005). Inflammation induced lymphangiogenesis in the cornea arises from CD11b-positive macrophages.. J Clin Invest.

[24] Capla JM, Ceradini DJ, Tepper OM, Callaghan MJ, Bhatt KA (2006). Skin graft vascularization involves precisely regulated regression and replacement of endothelial cells through both angiogenesis and vasculogenesis.. Plast Reconstr Surg.

[25] Saaristo A, Tammela T, Timonen J, Yla-Herttuala S, Tukiainen E (2004). Vascular endothelial growth factor-C gene therapy restores lymphatic flow across incision wounds.. Faseb J.

[26] Kerjaschki D (2005). The crucial role of macrophages in lymphangiogenesis.. J Clin Invest.

[27] Saaristo A, Tammela T, Farkkila A, Karkkainen M, Suominen E (2006). Vascular endothelial growth factor-C accelerates diabetic wound healing.. Am J Pathol.

[28] Cursiefen C, Chen L, Borges LP, Jackson D, Cao J (2004). VEGF-A stimulates lymphangiogenesis and hemangiogenesis in inflammatory neovascularization via macrophage recruitment.. J Clin Invest.

[29] Maruyama K, Ii M, Cursiefen C, Jackson DG, Keino H (2005). Inflammation-induced lymphangiogenesis in the cornea arises from CD11b-positive macrophages.. J Clin Invest.

